# Virome antigens as triggers for immune recognition of mutant clones in normal tissues

**DOI:** 10.1172/jci.insight.203484

**Published:** 2026-05-05

**Authors:** Natalie E. Andresen, Heehwa G. Son, Joongho J. Joh, Shadmehr Demehri

**Affiliations:** 1 Arthur and Sandra Irving Center for Cancer Immunology, Krantz Family Center for Cancer Research, and; 2 Cutaneous Biology Research Center, Department of Dermatology, Massachusetts General Hospital and Harvard Medical School, Boston, Massachusetts, USA.; 3 Department of Medicine, University of Louisville School of Medicine, Louisville, Kentucky, USA.; 4 Brown Cancer Center, University of Louisville Health Sciences Center, Louisville, Kentucky, USA.; 5 Department of Dermatology, Massachusetts General Hospital and Harvard Medical School, Boston, Massachusetts, USA.

**Keywords:** Dermatology, Oncology, Virology, Adaptive immunity, Skin cancer

## Abstract

Human virome antigens, but not tumor-associated antigens or neoantigens, may be essential to initiate the recognition of the carcinogen-induced mutant clones within a normal tissue.

**To the Editor:** The contribution of tumor-associated antigens and neoantigens to T cell recognition of cancer cells and immunotherapy response is well established (1). However, the antigens that can trigger T cell recognition of mutant cells and clones within normal tissues remain unknown. Ultraviolet (UV) radiation induces mutant keratinocyte clones in the epidermis, which can progress to keratinocyte carcinoma (KC) (2). Immunosuppressed individuals are at significantly higher KC risk due to compromised T cell immunity (2). One possibility is that neoantigens trigger CD8^+^ T cell recognition of mutant clones in the epidermis since UV induces a high mutational burden in keratinocytes. However, we propose that immune control of mutant clones is initiated by T cells detecting viral antigens presented by mutant cells. Our previous work has demonstrated that mice colonized with mouse papillomavirus (MmuPV1), to model human papillomavirus (HPV) skin colonization, are protected from UV-induced skin cancer through CD8^+^ T cell control of mutant-p53 clones (3, 4).

To explore whether neoantigens or viral antigens are the initial triggers for T cell recognition of mutant clones in the epidermis, we compared somatic mutational burden in nonlesional back skin of immunocompetent SKH-1 mice after the UV skin carcinogenesis protocol with that in sun-exposed human facial skin (Figure 1A) (5). UV signature mutations were similarly dominant in mouse and human skin, while mouse skin displayed a higher mutational burden (Figure 1, B and C). Despite a high mutational burden, the number of skin tumors did not significantly increase after CD8^+^ T cell depletion in UV-treated SKH-1 mice (Figure 1, D–F). MmuPV1-infected mice subjected to the same UV protocol showed a comparable mutational landscape, but markedly increased epidermal CD8^+^ T cells compared with uninfected mice (Figure 1, G–J). Importantly, CD8^+^ T cell depletion in MmuPV1-infected mice significantly increased UV-induced skin tumor development (Figure 1, K–M).

Given the high mutational burden in UV-treated mouse skin, these results suggest that in the absence of papillomavirus infection, neoantigens generated by UV-induced mutations are not sufficient to trigger CD8^+^ T cell immunity to prevent KC development. However, T cells’ ability to control mutant clones in the epidermis is established upon papillomavirus colonization, which phenocopies the ubiquitous presence of cutaneotropic HPVs as prominent members of the human skin virome (3, 4). We propose that previously acquired virus-specific memory T cells in the skin are essential in initiating the recognition of virus-colonized mutant clones. Subsequently, epitope spreading to neoantigen-specific T cells may occur, leading to enhanced cancer immunosurveillance. While certain β-HPV types, such as HPV8, have been reported to promote oncogenesis by interfering with NOTCH and TGF-β signaling (6), our model underscores the virus’s role as a potent immune trigger, independent of its direct oncogenic potential. This is supported by our previous observations that β-HPV is effectively regulated by the host immune system (3, 4). Specifically, β-HPV E6/7 transcripts are more abundant in normal sun-exposed skin compared with actinic keratosis and cutaneous squamous cell carcinoma in immunocompetent patients. Consistently, CD3^+^ and CD8^+^ T cells are enriched within mutant-p53 clones in human facial skin, particularly those exhibiting high β-HPV activity, compared with non-clonal or sun-protected skin. These findings suggest early immune targeting of virus-infected mutant clones, while loss of viral antigen expression contributes to immune evasion and tumor development. Thus, we propose that the host immune system actively utilizes the virome to initiate immunosurveillance in order to maintain tissue homeostasis and prevent the progression of carcinogen-induced mutant clones into malignancy.

## Conflict of interest

SD is an inventor on a filed patent for the development of T cell–directed anti-cancer vaccines against commensal viruses (PCT/US2019/063172). All other authors declare no conflict of interest. The interests of SD are reviewed and managed by Massachusetts General Hospital and Mass General Brigham HealthCare in accordance with their conflict-of-interest policies.

## Funding support

This work is the result of NIH funding, in whole or in part, and is subject to the NIH Public Access Policy. Through acceptance of this federal funding, the NIH has been given the right to make the work publicly available in PubMed Central.

- Career Award for Medical Scientists from the Burroughs Wellcome Fund (to SD).
- LEO Foundation Award LF-AW_RAM-22-400154 (to SD).
- NIH grant R01 CA251755 (to JJJ and SD).

## Supplementary Material

Supplemental data

Supporting data values

## Figures and Tables

**Figure 1:**
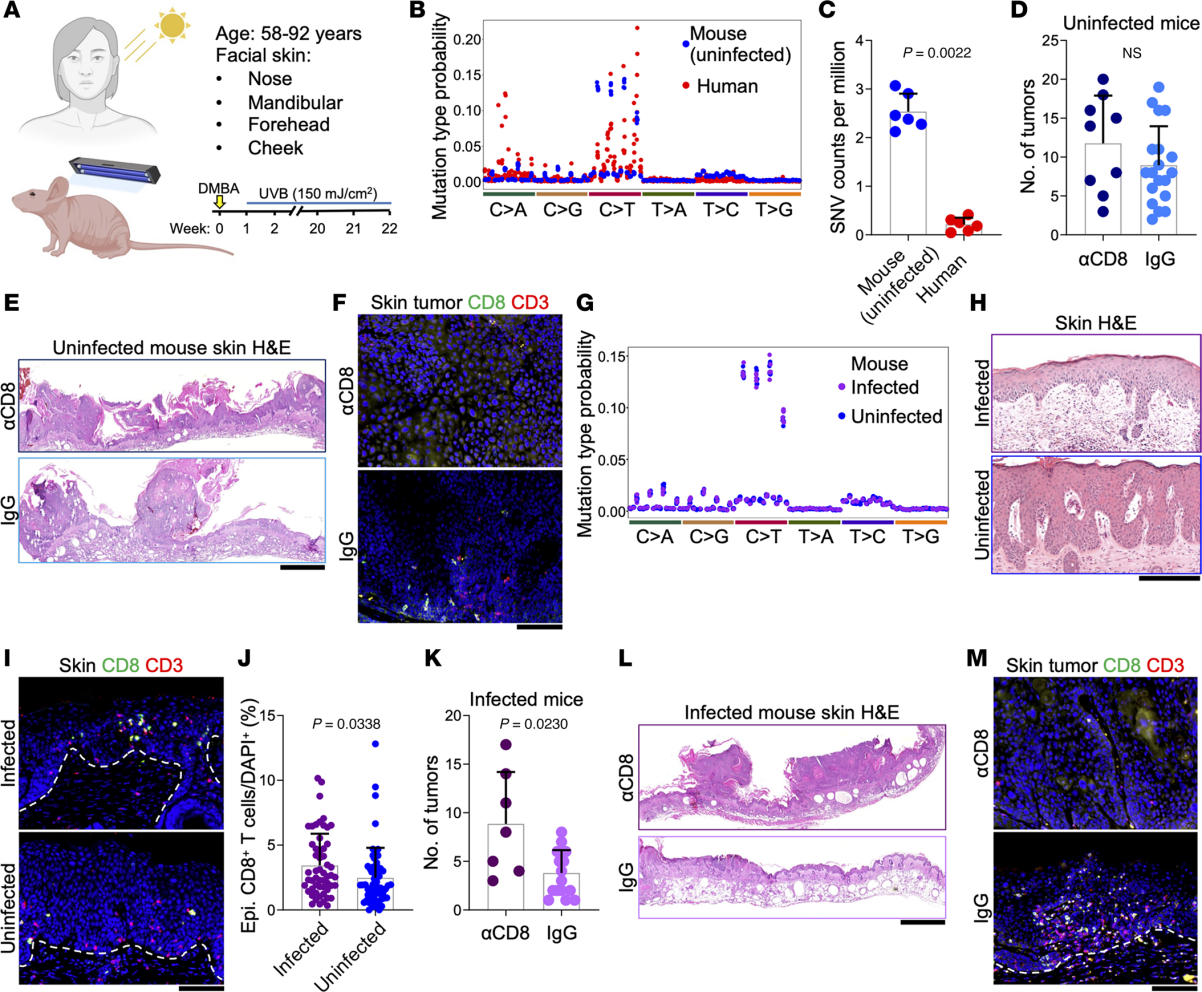
Papillomavirus colonization, not high mutational burden, dictates CD8^+^ T cell protection against UV-induced skin cancer development. (**A**) Diagram of sun-exposed human skin and SKH-1 mice treated with UV skin carcinogenesis protocol. DMBA, 7,12-dimethylbenz[*a*]anthracene. (**B** and **C**) Mutational signatures (**B**) and SNV counts per million (**C**) in human facial skin (*n* = 6) and UV-treated uninfected SKH-1 mouse back skin (*n* = 6). SNV, single-nucleotide variant. (**D**) Tumor burden at week 22 after UV treatment in CD8^+^ T cell–depleted (*n* = 9) versus IgG control (*n* = 19) uninfected SKH-1 mice. (**E** and **F**) Representative images of H&E-stained back skin (**E**) and CD8/CD3 immunofluorescently stained tumors (**F**) of UV-treated uninfected SKH-1 mice given anti-CD8 antibody (αCD8) or IgG control. (**G**) Mutational signatures in MmuPV1-infected (infected) and uninfected SKH-1 mice (*n* = 6 per group). Mutation type probabilities for uninfected mice are shown in **B**. Total mutation burden is comparable between UV-treated infected and uninfected mouse skin (3). (**H** and **I**) Representative images of H&E-stained (**H**) and CD8/CD3 immunofluorescently stained (**I**) back skin of MmuPV1-infected versus uninfected SKH-1 mice. (**J**) Quantified epidermal CD8^+^ T cells in MmuPV1-infected versus uninfected SKH-1 skin after UV treatment (*n* = 6 mice per group). T cells were counted in up to 10 randomly selected high-power fields (original magnification, ×200) per mouse. (**K**) Tumor burden at week 22 after UV treatment in CD8^+^ T cell–depleted (*n* = 7) versus IgG control (*n* = 16) MmuPV1-infected SKH-1 mice after UV treatment. (**L** and **M**) Representative images of H&E-stained back skin (**L**) and CD8/CD3 immunofluorescently stained tumors (**M**) of UV-treated MmuPV1-infected SKH-1 mice given αCD8 or IgG control. Dashed lines mark the epidermal basement membrane. Significance assessed by 2-tailed Mann-Whitney *U* test (**C**, **D**, and **K**) or 2-tailed unpaired *t* test (**J**)*.* NS, not significant. Bar graphs show mean + SD. Scale bars: 1 mm (**E** and **L**) and 100 μm (**F**, **H**, **I**, and **M**).

## Version 1 05/05/2026

In-Press Preview

## Version 2 06/22/2026

Electronic publication
