# Supplementary material for: Virome antigens as triggers for immune recognition of mutant clones in normal tissues
Source: JCI Insight. 2026 May 5;11(12):e203484. doi: 10.1172/jci.insight.203484 (PMC13313533; doi:10.1172/jci.insight.203484)
Supplement: Supplemental data [file jciinsight-11-203484-s002.pdf]

Andresen et al.

Supplementary Appendix:

Table of Contents

Supplementary Methods

Supplementary Text

References

Supplementary Methods

Sex as a biological variable.
Female mice were used to avoid the potential for aggressive behavior and associated skin wounds in males, which could have confounded the experimental outcomes.

Animal studies
All mice were handled in accordance with the University of Louisville and Massachusetts General Hospital animal care regulations. Age-matched female SKH-1 Elite mice (Charles River Laboratories, catalog no. 477) were used for all experiments.

UV skin treatment
Preparation of MmuPV1 and virus-like particle (VLP) followed previously published methods (1). SKH-1 mice were anesthetized with isoflurane (Baxter Healthcare, Deerfield, IL; catalog no. 10019-360-40) for the abrasion of the dorsal skin. Abrasion was performed using a nail-buffer sanding block (Karlash). A total of 1 × 10^{10} VGE of MmuPV1 (i.e., infected mice) or 78 µg of MmuPV1 VLP in 20 µL PBS (i.e., uninfected mice) was then applied to the prepared skin surface and spread uniformly. For analgesia, mice received a subcutaneous dose of meloxicam (0.5 mg/kg; Boehringer Ingelheim Vetmedica) or 200 µg Rimadyl/Carprofen (Zoetis, catalog no. 024751), with an additional dose administered the following day. After establishing viral presence, animals were treated at Week 0 with 50 µg DMBA dissolved in 200 µL acetone applied to the dorsal skin. One week later, mice underwent UVB exposure (150 mJ/cm^2) three times weekly. SKH-1 mice continued this regimen for up to 22 weeks. UVB irradiation was delivered at 302 nm with a UVP XX-15MR Bench Lamp (Analytik Jena; catalog no. 95004208). Output was verified using a digital light meter (International Light Technologies, catalog no. ILT1400A).

Exome-seq and analysis
Mouse exome-sequencing datasets were retrieved from the NCBI Sequence Read Archive (SRA) under BioProject PRJNA954057 (1). Human exome-sequencing datasets were obtained from the NCBI SRA under BioProject PRJNA753012 (2). Sequencing reads were aligned to the mm39 mouse reference or hg38 human genome reference using the Burrows–Wheeler Aligner (BWA) software (3) for mouse and human samples, respectively. Variant discovery, including identification of single-nucleotide polymorphisms and insertion/deletion events, was performed with GATK (4). Variant Call Format (VCF) outputs were subsequently annotated through SnpEff (5). Analysis of mutational patterns and extraction of mutation signatures were conducted in R using the signeR (6) package.

Histology and immunofluorescence (IF) staining
Mouse tissue samples were harvested and preserved in 4% paraformaldehyde (Millipore Sigma, catalog no. 441244) at 4°C overnight. Then, samples were passed through graded ethanol solutions (30%, 50%, 70%) for dehydration and subsequently processed. They were embedded in paraffin the following day. Paraffin blocks were sectioned at 5 µm, mounted onto glass slides, and then paraffin was removed with xylene (Millipore Sigma, catalog no. 534056), and sections were prepared for H&E staining. For immunofluorescence, paraffin sections were cleared with

xylene, rehydrated, and subjected to antigen retrieval as previously described. PBS containing 0.1% Tween-20 was used for washing, after which sections were incubated in 5% goat serum for one hour to block nonspecific binding. Primary antibodies (Rabbit anti-mouse CD8, Cell Signaling Technology, catalog no. 98941, clone D4W2Z and rat anti-mouse CD3, Abcam, catalog no. ab11089, clone CD3-12) were applied and left to bind overnight at 4°C. The next day, fluorophore-linked secondary antibodies (Goat anti-Rabbit IgG, Alexa Fluor® 488 conjugate, Thermo Fisher Scientific, catalog no. A-11034 and Goat anti-Rat IgG, Alexa Fluor® 568 conjugate, Thermo Fisher Scientific, catalog no. A-11077) were added and allowed to incubate for 30 minutes at 37°C. Nuclear staining was performed using DAPI (Thermo Fisher Scientific, catalog no. D3571) for 5 minutes at room temperature. Slides were then washed and coverslipped with Fluoroshield mounting medium (Millipore Sigma, catalog no. F6182). Fluorescent images were acquired using a ZEISS Axio Observer Z1 microscope (Zeiss).

$CD8^+$ T cell depletion and IR700.

IR700 conjugates were generated by labeling anti-CD8β antibodies (rat anti–mouse CD8β, Lyt 3.2; clone 53-5.8; BioXCell, catalog no. BE0223) and a rat IgG isotype control (Millipore Sigma, catalog no. I4131) with the IRDye 700DX Protein Labeling Kit (LI-COR Biosciences, catalog no. 928-38046), following the kit's instructions. During the 15th week of UV exposure, mice were given an intraperitoneal dose of 750 µg of either anti-CD8β–IR700 or IgG–IR700. After a 24 hour interval, animals were exposed to near-infrared light (690 nm) at 50 J/cm^2 using an infrared illuminator (Marubeni, catalog no. L690D-66-60-550). From week 16 onward, mice received weekly intraperitoneal injections of 250 µg of the respective IR700-labeled antibody, each followed by a 75 J/cm^2 NIR irradiation session for a total duration of 5 weeks.

Statistics.

Mean and standard deviation (s.d.) are indicated by bar graphs and dot plots. To calculate p values, two-tailed Mann-Whitney U tests or two-tailed unpaired t-tests were used, considering $p < 0.05$ as significant. Individual data points are provided in the accompanying Supporting Data Values XLS file.

Acknowledgments:
We thank Dat Thinh Ha from the University of Louisville for assistance with mouse experiments.

Study approval:
All animal studies were reviewed and approved by the University of Louisville and Massachusetts General Hospital Institutional Animal Care and Use Committee.

Data availability:
Data related to this paper may be requested from the corresponding author.

Author contributions statement:
S.D. conceived and directed the study. N.E.A. and H.G.S. performed experiments. N.E.A., H.G.S., and S.D. analyzed data and wrote the manuscript. J.J.J. provided intellectual input. All authors reviewed and approved the manuscript.

Competing financial interests:
S.D. is an inventor on a filed patent for the development of T cell-directed anti-cancer vaccines against commensal viruses (PCT/US2019/063172). Other authors declare no conflict of interest. The interests of S.D. are reviewed and managed by Massachusetts General Hospital and Mass General Brigham HealthCare in accordance with their conflict-of-interest policies.

References

1. Son HG et al. Commensal papillomavirus immunity preserves the homeostasis of highly mutated normal skin. *Cancer cell*. 2025;43(1):36–48.e10.
2. Kim YS, Bang CH, Chung YJ. Mutational Landscape of Normal Human Skin: Clues to Understanding Early-Stage Carcinogenesis in Keratinocyte Neoplasia. *J Invest Dermatol*. 2023;143(7):1187-1196.e9.
3. Li H, Durbin R. Fast and accurate long-read alignment with Burrows-Wheeler transform. *Bioinformatics*. 2010;26(5):589-595.
4. DePristo MA et al. A framework for variation discovery and genotyping using next-generation DNA sequencing data. *Nat Genet*. 2011;43(5):491-498.
5. Cingolani P et al. A program for annotating and predicting the effects of single nucleotide polymorphisms, SnpEff: SNPs in the genome of Drosophila melanogaster strain w1118; iso-2; iso-3. *Fly (Austin)*. 2012;6(2):80-92.
6. Rosales RA, Drummond RD, Valieris R, Dias-Neto E, da Silva IT. signeR: an empirical Bayesian approach to mutational signature discovery. *Bioinformatics*. 2017;33(1):8-16.
